# 

**Published:** 1882-01

**Authors:** 


					﻿Whole Number,
CCCCXLj^.-*^
Jan., 1882.
Number 1,
Vol. XLIV.
THE
CHICAGO
MEDICAL
J ournal and Examiner
EDITORS:
N. S. DAVIS. M.D., LL.D. JAS. NEVINS HYDE, A.M., M.D.
DANIEL R. BROWER, A.M. M.D.
CHICAGO:
PUBLISHED BY THE CHICAGO MEDICAL PHESS ASSOCIATION,
188 Clark Street.
[^'Read the Notice of this Journal amonp Advertisements.
				

